# Ultrasound-mediated blood–brain barrier opening uncovers an intracerebral perivenous fluid network in persons with Alzheimer’s disease

**DOI:** 10.1186/s12987-023-00447-y

**Published:** 2023-06-16

**Authors:** Rashi I. Mehta, Jeffrey S. Carpenter, Rupal I. Mehta, Marc W. Haut, Peng Wang, Manish Ranjan, Umer Najib, Pierre-François D’Haese, Ali R. Rezai

**Affiliations:** 1 Department of Neuroradiology, West Virginia University, 1 Medical Center Dr, Morgantown, WV 26506 USA; 2 Department of Neuroscience, West Virginia University, Morgantown, WV 26506 USA; 3 Rockefeller Neuroscience Institute, West Virginia University, Morgantown, WV 26506 USA; 4 Rush Alzheimer’s Disease Center, Rush University Medical Center, Chicago, IL 60612 USA; 5 Department of Pathology, Rush University Medical Center, Chicago, IL 60612 USA; 6 Department of Behavioral Medicine and Psychiatry, West Virginia University, Morgantown, WV 26506 USA; 7 Department of Neurosurgery, West Virginia University, Morgantown, WV 26506 USA; 8 Department of Neurology, West Virginia University, Morgantown, WV 26506 USA

**Keywords:** Alzheimer's disease, Blood–brain barrier opening, Focused ultrasound, Glymphatic efflux, Interstitial efflux, Neurofluid, Perivenous exudate, Perivenous space

## Abstract

**Background:**

Focused ultrasound (FUS)-mediated blood–brain barrier (BBB) opening is under investigation as a therapeutic modality for neurodegeneration, yet its effects in humans are incompletely understood. Here, we assessed physiologic responses to FUS administered in multifocal brain sites of persons with Alzheimer’s disease (AD).

**Methods:**

At a tertiary neuroscience institute, eight participants with AD (mean age 65, 38% F) enrolled in a phase 2 clinical trial underwent three successive targeted BBB opening procedures at 2 week intervals using a 220 kHz FUS transducer in combination with systemically administered microbubbles. In all, 77 treatment sites were evaluated and encompassed hippocampal, frontal, and parietal brain regions. Post-FUS imaging changes, including susceptibility effects and spatiotemporal gadolinium-based contrast agent enhancement patterns, were analyzed using serial 3.0-Tesla MRI.

**Results:**

Post-FUS MRI revealed expected intraparenchymal contrast extravasation due to BBB opening at all targeted brain sites. Immediately upon BBB opening, hyperconcentration of intravenously-administered contrast tracer was consistently observed around intracerebral veins. Following BBB closure, within 24–48 h of FUS intervention, permeabilization of intraparenchymal veins was observed and persisted for up to one week. Notably, extraparenchymal meningeal venous permeabilization and associated CSF effusions were also elicited and persisted up to 11 days post FUS treatment, prior to complete spontaneous resolution in all participants. Mild susceptibility effects were detected, however no overt intracranial hemorrhage or other serious adverse effects occurred in any participant.

**Conclusions:**

FUS-mediated BBB opening is safely and reproducibly achieved in multifocal brain regions of persons with AD. Post-FUS tracer enhancement phenomena suggest the existence of a brain-wide perivenous fluid efflux pathway in humans and demonstrate reactive physiological changes involving these conduit spaces in the delayed, subacute phase following BBB disruption. The delayed reactive venous and perivenous changes are consistent with a dynamic, zonal exudative response to upstream capillary manipulation. Further preclinical and clinical investigations of these FUS-related imaging phenomena and of intracerebral perivenous compartment changes are needed to elucidate physiology of this pathway as well as biological effects of FUS administered with and without adjuvant neurotherapeutics.

*Trial registration*: ClinicalTrials.gov identifier: NCT03671889, registered 9/14/2018

**Supplementary Information:**

The online version contains supplementary material available at 10.1186/s12987-023-00447-y.

## Introduction

Alzheimer’s disease (AD) is the third leading cause of death among elderly persons in the United States and its prevalence is increasing, yet treatment options remain limited [1, 2]. Novel neurotherapeutic approaches and techniques for this disease are urgently needed [3]. Given promising effects in preclinical models, blood–brain barrier (BBB) opening with focused ultrasound (FUS) is currently being explored as a potential treatment strategy for neurodegenerative disorders, including AD [4–10]. This nonsurgical intervention combines transcranially directed acoustic energy with an intravenously-administered microbubble contrast agent to transiently and reversibly enhance brain capillary permeability. FUS-mediated BBB opening has emerged as a feasible means of directed intracerebral drug delivery [4, 11]. This procedure has additionally been shown to induce neurogenesis, mitigate AD pathology, and improve cognitive behavior in animal models, with effects linked to a transient sterile intraparenchymal neuroinflammatory response [6, 10, 12–14].

Despite extensive preclinical analyses, knowledge on FUS-mediated BBB opening effects in live humans remains limited. Notably, species-specific differences in FUS effects have been reported due to structural and physiological variations of the brain, cranium, and cerebrovasculature across small and large animals [13]. Effects of FUS-mediated BBB opening and knowledge on how these may be mechanically or pharmacologically modulated to achieve neuroprotection in humans are poorly understood [15]. Although early phase clinical trial data suggest safety and feasibility of this technique [16–20] additional data are needed in humans to characterize the underlying physiological responses to this intervention, including in persons with AD. Elucidation of clinical effects could ultimately lead to refined and augmented approaches for treating neurodegeneration using FUS. Here, we explored biological responses following FUS-mediated BBB opening in the frontal, parietal, and medial temporal lobes of persons with early AD enrolled in a phase 2 clinical trial at a tertiary academic facility using serial contrast-enhanced 3.0-Tesla MRI and report findings in 8 trial participants.

## Materials and methods

### Clinical trial and study participants

A prospective phase 2 clinical trial was initiated at Rockefeller Neuroscience Institute, a tertiary academic neuroscience center, according to a protocol approved by the U.S. Food and Drug Administration and local institutional review board (ClinicalTrials.gov identifier, NCT03671889). This ongoing trial is sponsored by Insightec (Haifa, Israel). Informed consent was obtained from each participant at the time of trial enrollment.

**Fig. 1:**
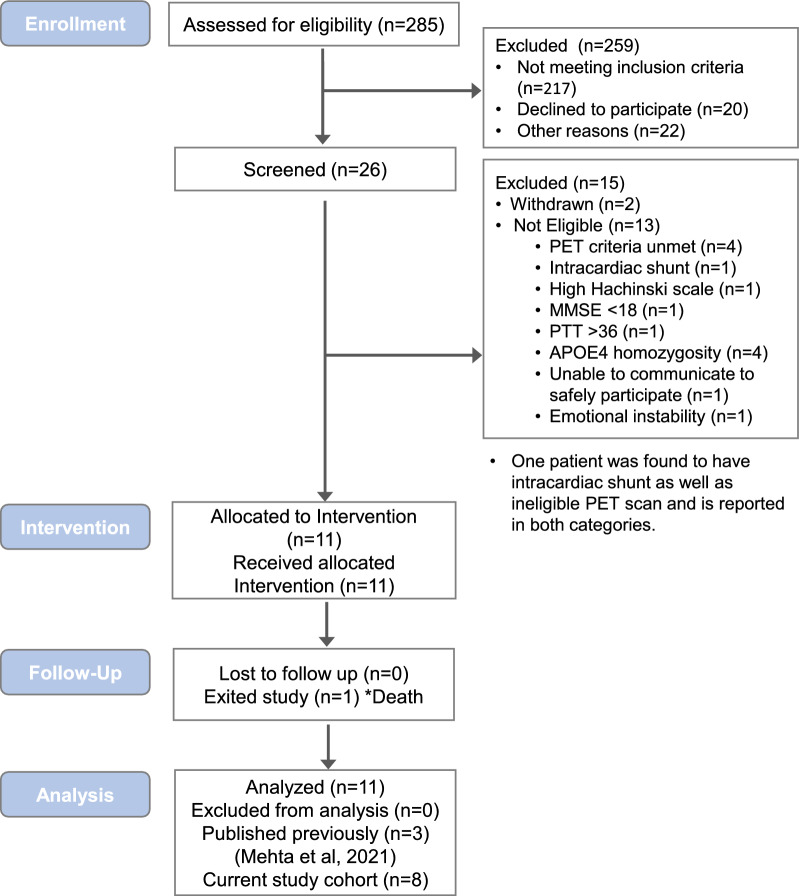
Clinical trial flow diagram. Imaging findings of participants 1–3 are published previously.^20^ Participants number 4 through 11 (ClinicalTrials.gov identifier: NCT03671889) are included in the current study cohort. *Death due to pancreatic adenocarcinoma in one participant during study follow-up (44 weeks after study enrollment; 36 weeks after completion of FUS treatment) was unrelated to the FUS intervention

Eight participants (mean age 65 years; 38% female) enrolled after meeting study eligibility requirements are included in the current study cohort (Fig. 1). Subjects were enrolled consecutively and represent the fourth through eleventh clinical trial participants at our institution. Neuroimaging outcomes of the first three trial participants who underwent FUS following a slightly different methodological technique, were recently published in a separate report [20]. Inclusion criteria for study enrollment included mild AD diagnosed with National Institute on Aging–Alzheimer’s Association criteria [21], fluorine [18F] florbetaben PET positivity for amyloid-β plaques, and lack of other known central nervous system disease. Additional inclusion and exclusion criteria are summarized in Additional file 1. Participant accrual for this trial began in September 2018. Subjects of the current analysis underwent FUS-mediated BBB opening procedures between November 2019 and July 2022. Patient enrollment and follow-up are ongoing.

### MRI-guided focused ultrasound protocol

Baseline pre- and post-contrast 3.0-Tesla MRI and 18F-florbetaben PET-CT data were acquired within one month before treatment. Multiple target volumes were selected prior to sonication based on individual anatomy and safety considerations at baseline MRI, as well as amyloid-β plaque burden assessed on the pre-treatment PET scan. Individualized treatment plans were created using Exablate software (Insightec, Haifa, Israel; v7.40 and v7.43).

**Fig. 2:**
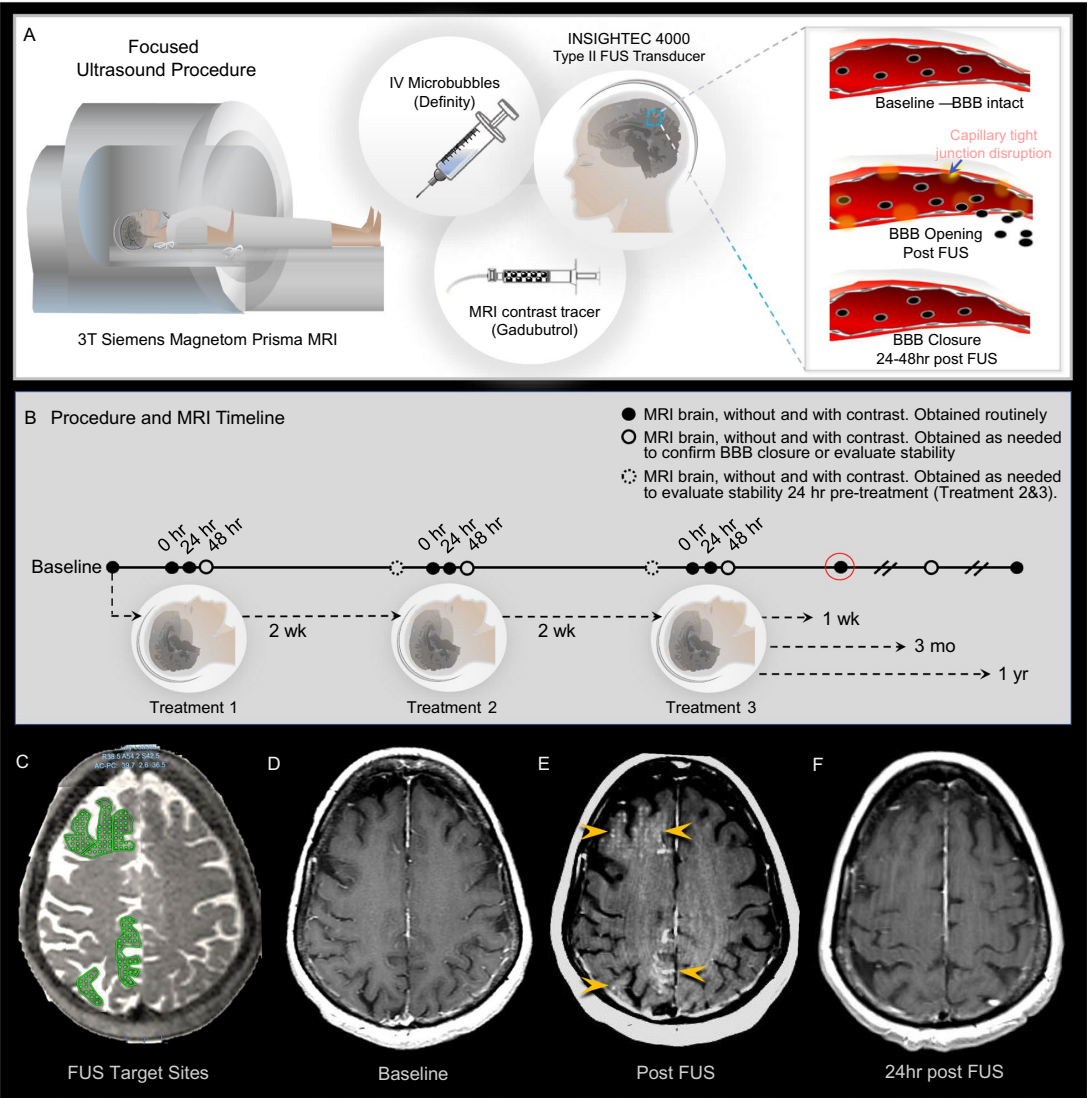
FUS procedure for targeted transient blood–brain barrier opening. The study was conducted using the Insightec Type II FUS transducer in combination with Definity® microbubbles **A**. Imaging was acquired using a 3-Tesla Siemens MRI scanner and gadobutrol contrast tracer in 8 volunteer subjects with early Alzheimer’s disease. The treatment and MRI timeline is shown **B**. FUS target sites within the frontal and parietal lobes are shown in green **C**. MRI scans show blood–brain barrier (BBB) opening and closure within these targeted brain volumes in a 73-year-old woman with Alzheimer’s disease **D**–**F**. Post-contrast T1 weighted images, at baseline **D**, immediately after FUS treatment **E**, and 24 h after treatment **F** show contrast tracer extravasation into parenchyma (arrowheads, **E**) due to focal BBB opening at the prescribed treatment sites. The BBB is closed at 24 h post FUS intervention **F**, with no contrast enhancement seen at target sites following repeat gadobutrol contrast tracer administration. *BBB* blood–brain barrier, *Hr* hour, *IV* intravenous, *Mo* month, *Tx* treatment, *Wk* week, *Yr* year

Each study subject underwent three successive sonication sessions (Fig. 2A), administered at 2 week intervals (Fig. 2B). The non-dominant (right) hippocampus and entorhinal cortex were targeted in subject 1, whereas the frontal and parietal lobes were sonicated in addition to the medial temporal lobes in subjects 2–8 (Fig. 2C, Table 1). In some patients, volumes in bilateral frontal and/or parietal lobes were targeted. Each sonication session consisted of stereotactic headframe placement, with administration of local anesthesia, or use of a dental mold assembly. Participants were placed in the supine position, with the ExAblate 4000 low-frequency type 2 system hemispherical transducer (Insightec, Haifa, Israel), consisting of 1024 phased-array elements with a frequency of 220 kHz, positioned over the head. The FUS transducer was integrated with a clinical Siemens 3.0-Tesla Magnetom Prisma MRI unit (Siemens, Erlangen, Germany). Following initiation of intravenous infusion of perflutren microbubble contrast material (Definity^®^; Lantheus Medical Imaging), target volumes were sonicated (Fig. 2).

**Table 1:** Characteristics of 8 participants with AD.

Subject no	Age (years)	Sex	MMSE score	ADAS-COG score	ApoE	Brain regions treated by FUS
1	68	M	22	19	ε3/ε3	Medial temporal
2	54	F	19	24	ε3/ε3	Medial temporal, Parietal
3	63	M	24	17	ε3/ε4	Medial temporal, Parietal, Frontal
4	70	M	22	15	ε3/ε4	Medial temporal, Parietal, Frontal
5	76	F	25	17	ε3/ε4	Medial temporal, Parietal, Frontal
6	67	F	21	19	ε3/ε4	Medial temporal, Parietal, Frontal
7	57	M	20	19	ε3/ε3	Medial temporal, Parietal, Frontal
8	68	M	22	14	ε2/ε4	Medial temporal, Parietal, Frontal

*ADAS-COG* Alzheimer’s disease assessment scale—cognitive subscale, *ApoE* apolipoprotein *E MMSE* mini-mental state examination

Each individually sonicated treatment target consisted of a variable number of sonication spots and brain volume. Spacing between sonication spots was set at 3 mm and the largest individually targeted volume consisted of 32 sonication spots. Three to four target volumes were treated in the medial temporal lobes of each subject. Six to 11 targets were treated in each individual’s parietal lobes. Five to 11 targets were treated in the frontal lobes. The total treatment volume varied from 1 to 2 cc in the hippocampus/entorhinal cortex, 7 to 31 cc in the parietal lobes, and 7 to 22 cc in the frontal lobes for each subject.

The sonication repetition time was set at 1 s and each sonication spot received ultrasound power with 5 ms duration and evenly distributed across the target in one repetition. The cavitation dose, which is derived from the area under the curve of selected sub-harmonic microbubble acoustic response, was selected. The prescribed cavitation dose for targets at hippocampi varied from 1.8 to 2.2 V·s and at parietal and frontal lobes varied from 0.7 to 1.5 V·s. Based on the cavitation dose, the system controller automatically adjusts in real time the acoustic power to optimally cavitate circulating microbubbles. The treatment time for each target is typically 90 s. Sonication was repeated at certain subspot locations in some patients, due to undertreatment caused by local inhomogeneity, to achieve the desired cumulative cavitation dose at targeted tissue volumes.

The administration of perflutren (Definity^®^, Lantheus Medical Imaging) microbubble contrast was via drip infusion for all but the first subject of this series. The initial subject received activated Definity^®^ using a dilute bolus technique. Dosing for the dilute bolus method was 4 µl/kg per sonication (maximum per session was 20 µl/kg). Drip infusion dosing for subjects 2–6 was 1.3 mL of activated perflutren in 500 mL of preservative-free saline and administered at a rate of 4 mL/min. The final two subjects were given a 3 mL/min infusion of 1.5 mL of activated perflutren mixed with a 250 mL bag of preservative-free saline.

Brain target sites were treated again following the methods described above during subsequent second and third FUS sessions which were administered 2 and 4 weeks, respectively, after the initial procedure (Fig. 2B). Participants were monitored for 24 h after each therapy and, as of this writing, were clinically followed for up to 48 months after completion of therapy with formal neurologic and neuropsychologic assessments and periodic brain MRI exams.

### Posttreatment MRI protocol

Post-sonication MRI of the brain was conducted with the same clinical Siemens 3.0-Tesla Magnetom Prisma MRI unit (Siemens, Erlangen, Germany) by using a 20-channel head coil. Pre- and post-contrast MRI of the brain was performed immediately after completion of each sonication treatment session and again at 24 h following each FUS treatment. MRI was repeated at 48 h post intervention following one or more of the three treatment sessions in 6 out of 8 subjects (Fig. 2B, Table 1). Follow-up brain MRI was performed 1 week (on day 7 or 8) after completion of the final (third) treatment session for each participant, 5 weeks after initial FUS therapy (Fig. 2B). MRI sequences acquired included T2* gradient-echo sequences (GRE), susceptibility weighted imaging (SWI), diffusion, T2-weighted, pre- and post-contrast T2-weighted fluid-attenuated inversion recovery (FLAIR), and pre- and post-contrast T1-weighted sequences, with MRI sequence parameters detailed in Additional File 2. Timing of post-contrast sequences was uniform between study participants, with post-contrast T2-FLAIR imaging acquired at approximately 5 min post injection and post-contrast T1-weighted turbo spin echo sequences acquired at approximately 20 min post injection following all FUS treatment sessions. Gadobutrol (0.1 mmol per kilogram of body weight; Gadavist^®^; Bayer Healthcare Pharmaceuticals, Berlin, Germany; Molecular weight: ~600 Da), administered intravenously, was used as a contrast agent for all MRI studies.

### MRI analysis

Image analysis was independently conducted by two licensed and experienced, board-certified academic neuroradiologists with expertise in cerebrovascular imaging (RIM and JSC). Post-treatment MRI examinations were compared with pre- and post-contrast baseline MRI data. Analyses were conducted immediately after each MRI acquisition and included assessment for the presence versus absence and location of the following: Parenchymal enhancement, extraparenchymal enhancement, parenchymal hemorrhage, extraparenchymal hemorrhage, restricted diffusion, parenchymal and/or extraparenchymal T2-weighted FLAIR signal hyperintensity, and mass effect. The duration of these imaging effects was also assessed by analyzing serial MRI data. Both readers concurred on diagnostic findings and interpretations on all patients.

Post-treatment contrast enhancement data were correlated with cerebral angioarchitecture of treated subjects as well as that on MRI of a young reference patient (i.e., non-trial participant). SWI sequence analysis was performed to map intracerebral venous anatomy [22]. Treated brain regions were segmented on baseline and post-treatment MRI scans to assess for any difference in cortical brain volumes post FUS, compared to baseline. In addition, pre-contrast T1 signal intensity of treated volumes was assessed on post-treatment MRI and compared with baseline data and untreated brain regions. Maximal cerebrospinal fluid (CSF) effusion dimensions were measured by an experienced neuroradiologist (RIM) by identifying the largest confluent region of extraparenchymal signal hyperintensity on axial post-contrast T2-FLAIR sequences. CSF effusion measurements were acquired in treated frontal and parietal brain regions and conducted on MRI exams obtained on days 0, 1, and 2 (where available) following FUS intervention. Due to smaller target volumes in the hippocampus resulting in small volume effusions that were not routinely amenable to measurement in the axial plane, effusion dimensions were not measured in the medial temporal region.

### Quantification of MRI data

Maximum CSF effusion size values (averages following three FUS sessions) in the frontal and parietal regions at day 1 and day 2 were normalized to initial (day 0) effusion size and data were plotted as fold changes for each patient (Fig. 6D, E). Overall maximum effusion sizes at days 0, 1 and 2 following each individual FUS treatment were also plotted to display temporal effusion effects in the frontal and parietal regions for each subject (Fig. 6C). Additionally, correlation coefficient (R^2^) values were calculated to assess the association between effusion dimension (fold change at 24 h) with delivered FUS cavitation dose (Fig. 7). The percentage of subjects with specific signal change patterns on blood-sensitive susceptibility sequences and delayed effusion clearance was also analyzed (Fig. 6G, H).

To analyze differences in volume of the treated cortex, the volumetric T1-weighted images were processed using spatially localized atlas network tiles (SLANT) [23] to produce 132 brain segmentations for volumetric analysis (1 mm per segment). A series of one-way repeated measure ANOVAs was evaluated for each cortical region that was treated in 3 or more participants (hippocampus, entorhinal cortex, and precuneus) with analysis of baseline, 60 day and 1 year data.

### Histologic analysis of brain

Due to limited feasibility of perivenous space characterization by histology in live humans, postmortem tissues from elderly non-trial AD subjects and age-matched non-trial, non-AD decedents were examined histologically to further understand the MRI effects. Routine hematoxylin and eosin-stained slides corresponding to the superior frontal, parietal precuneus, and medial temporal brain regions were analyzed from three subjects per group. Brain samples sustained postmortem intervals of 48 h or less and were selected from hospital decedents without documented vascular diseases (including CAA), and with Braak stage of 0 and Thal stage of 0 (for non-AD group) or Braak stage of 4 and Thal stage of 3 or 4 (for AD group). Samples were prepared per standard protocol including 12 days of formalin fixation followed by paraffin embedment, six micron sectioning, and routine staining. Mean patient age was 65 years (age range, 64 to 67 years). Perivenous regions in cerebral cortex and subcortical white matter were evaluated on each slide by routine histologic and light microscopic techniques to assess the nature of perivenous microanatomy and potential flow paths visualized by in vivo MRI. Histological analysis was conducted by a licensed and experienced, board-certified academic neuropathologist expert on neurovascular anatomy and age-related brain changes.

## Results

### Study cohort

For this analysis, two hundred and eighty-five patients were assessed for eligibility (Fig. 1). Among them, 259 did not meet the study inclusion criteria (n = 217) or exhibited other reason(s) for nonenrollment (n = 42), and were excluded. The remaining 26 patients were screened. Among these, two declined to participate whereas 13 did not meet inclusion criteria due to negative amyloid PET results (n = 4), presence of an intracardiac shunt (n = 1), high Hachinski scale score (n = 1), MMSE (n = 1), PTT (n = 1), APOE Ɛ4 homozygosity (n = 4), inability to communicate (n = 1), and/or emotional instability (n = 1). Eleven patients were consecutively enrolled in the clinical trial at our institution. Imaging outcomes of 3 initial enrollees were reported previously [20]. The remaining enrolled participants are included in the current study cohort, which consists of 8 participants (mean age, 65 years; 38% women; Table 1).

### Transient reversible BBB opening is immediately achieved in multifocal brain regions

Immediately after completion of each FUS sonication, extravasation of intravenously administered contrast agent was identified within all treated brain volumes, indicating focal, reproducible, and spatially-precise BBB opening within the targeted frontal, parietal, and medial temporal lobes (Fig. 2). Pre-contrast MRI obtained 24 h post-intervention revealed complete spontaneous resolution of parenchymal contrast enhancement at all treated sites, with the return of baseline T1 signal intensity in all participants. No evidence of interstitial contrast enhancement was identified after repeated intravenous gadolinium-based contrast agent administration at 24–48 h post-intervention. These findings confirmed expected rapid clearance of interstitial contrast agent as well as BBB closure within 24–48 h of FUS intervention at all treated sites of each participant.

### Contrast concentrates along cerebral veins upon BBB opening

**Fig. 3:**
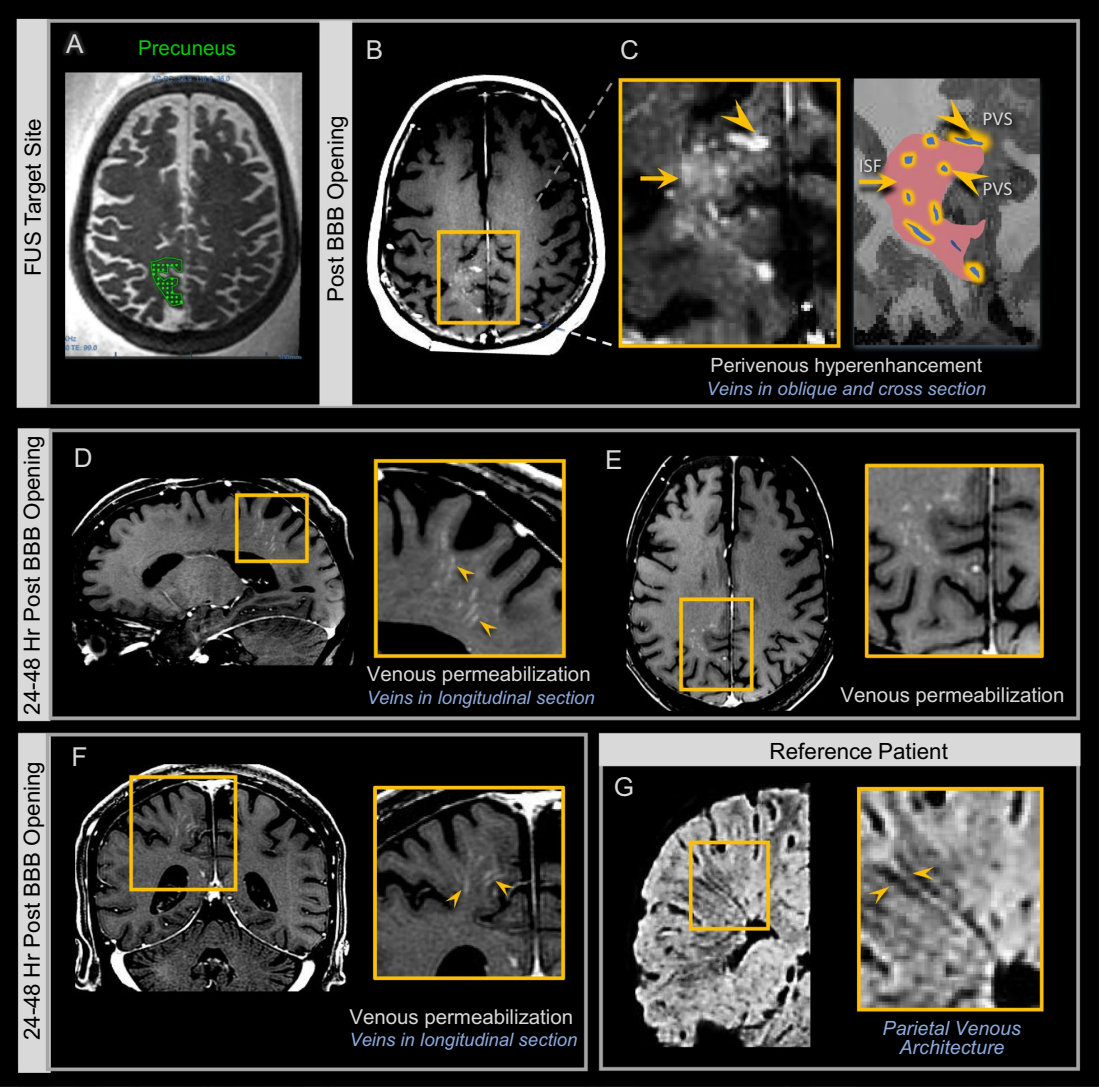
Perivenous hyperenhancement is identified upon BBB opening. FUS target map shown on axial T2-weighted sequence **A**. Post-contrast T1-weighted image immediately after FUS intervention shows contrast material extravasation due to BBB opening focally at the precuneus targeted site **B**. Note heterogeneous enhancement pattern within the targeted zone **C**, with contrast predominating along perivenous spaces (PVS), and less intense contrast enhancement in the remainder of the treatment zone interstitial fluid space (ISF). Pre-contrast MRI at 24 h post FUS showed complete clearance of contrast tracer (not shown). Post-contrast T1-weighted images in the same patient at 24–48 h revealed venous permeabilization manifesting as linear and curvilinear contrast enhancement within the FUS-treated zone, however there was no longer enhancement within the remaining interstitial fluid space **D**–**F**. This venous permeabilization enhancement corresponded anatomically to intraparenchymal and extraparenchymal parietal venous architecture, shown on SWI images **G**. See Additional files 3, 4 for additional images

Contrast enhancement within sonicated brain volumes following BBB opening was inhomogeneous. A representative image shows an example of the heterogeneous pattern of intracerebral contrast enhancement observed immediately upon FUS intervention (Fig. 3C). Intravenously administered contrast tracer distributed in higher concentrations around intraparenchymal veins, evidenced by differential signal hyperintensity in perivenous zones relative to remaining brain interstitial spaces (Fig. 3C). This differential enhancement phenomenon was observed in 8 of 8 treated individuals (100%), with variation in extent and degree. The contrast distribution resulted in linear and curvilinear hyperintensities that paralleled the superficial and deep venous structures within the sonicated frontal, parietal, and medial temporal lobes (Figs. 2, 3), and was also seen around extraparenchymal veins including the inferior sagittal sinus, as shown in Additional files 3, 4. This perivenous predominance of contrast was confirmed by venous mapping on SWI sequence analysis [22]. In some cases, a prominent grid-like enhancement pattern (as shown in the frontal lobe in Fig. 2E), corresponding to the prescribed FUS target spots (Fig. 2C), was also demonstrated.

### Intracerebral venous permeabilization is observed after BBB closure

**Fig. 4:**
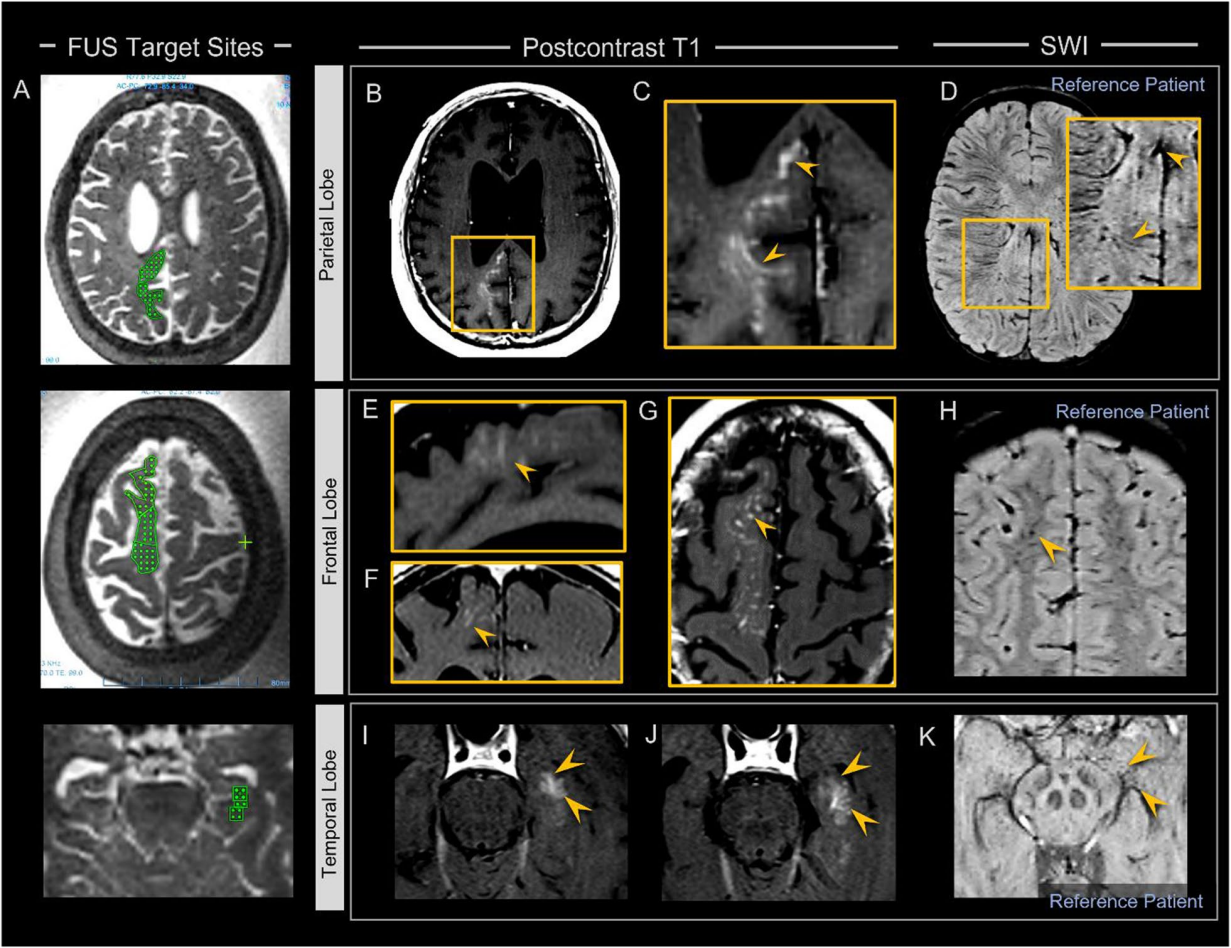
Venous permeabilization is observed multifocally within the brain following FUS mediated blood–brain barrier opening and persists beyond the time of BBB closure, for up to one week post FUS intervention. Parietal, frontal, and hippocampal FUS target sites are shown on T2-weighted images in three patients **A**. Post-contrast T1-weighted images show venous permeabilization manifesting as linear and curvilinear contrast enhancement within the FUS-treated zones within parietal, frontal, and hippocampal regions (arrowheads in **C**, **E**–**G, I, K**, respectively). This enhancement revealed branching configurations, and discrete arborized patterns, as shown in **I**, **J** extending to the deep venous system (i.e. basal vein of Rosenthal via hippocampal veins shown in **I**, **J**, **K**) and the dural venous sinuses (i.e. inferior sagittal sinus via posterior pericallosal vein, shown in **C**, **D**; see Additional files 3, 4 for additional images). Venous permeabilization enhancement corresponded anatomically to intraparenchymal and extraparenchymal parietal, frontal, and hippocampal venous architecture, as shown on susceptibility-weighted imaging (arrowheads, **D**, **H**, **K**)

**Fig. 5:**
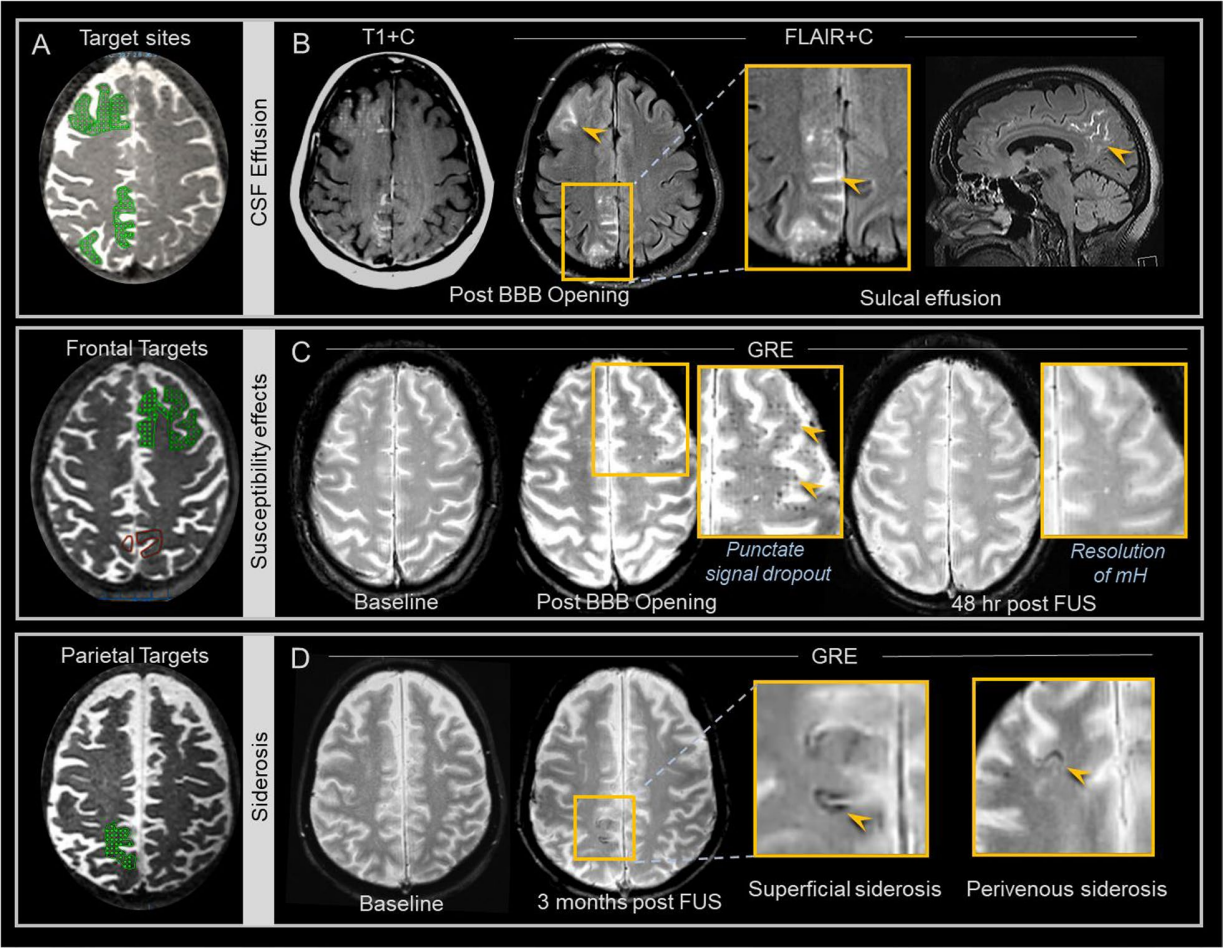
Cerebrospinal fluid effusions and susceptibility effects are observed after blood–brain barrier opening. Frontal and parietal FUS target sites are shown on T2-weighted images in three patients **A**. Post-contrast T1 (T1 + C) and T2-FLAIR (FLAIR + C) MRI images in a 73 year-old woman with Alzheimer’s disease (same patient shown in Fig. 2**B**) show CSF space hyperintensity (arrowheads), indicating CSF effusions, over frontal and parietal brain regions treated with FUS. GRE images in a 67 year-old woman with Alzheimer’s disease **C** show development of punctate foci of signal dropout at targeted frontal brain region (arrowheads) compared to baseline, with resolution of these signal changes by 48 h post FUS. The GRE signal changes did not always completely resolve, however were not associated with any clinical adverse effects in any subject. GRE images in a 54 year-old woman with Alzheimer’s disease **D** show development of superficial siderosis (arrowhead) along the surface of the treated parietal precuneus. Perivenous siderosis (arrowhead) is also shown following FUS in a 57 year-old male trial participant

**Fig. 6:**
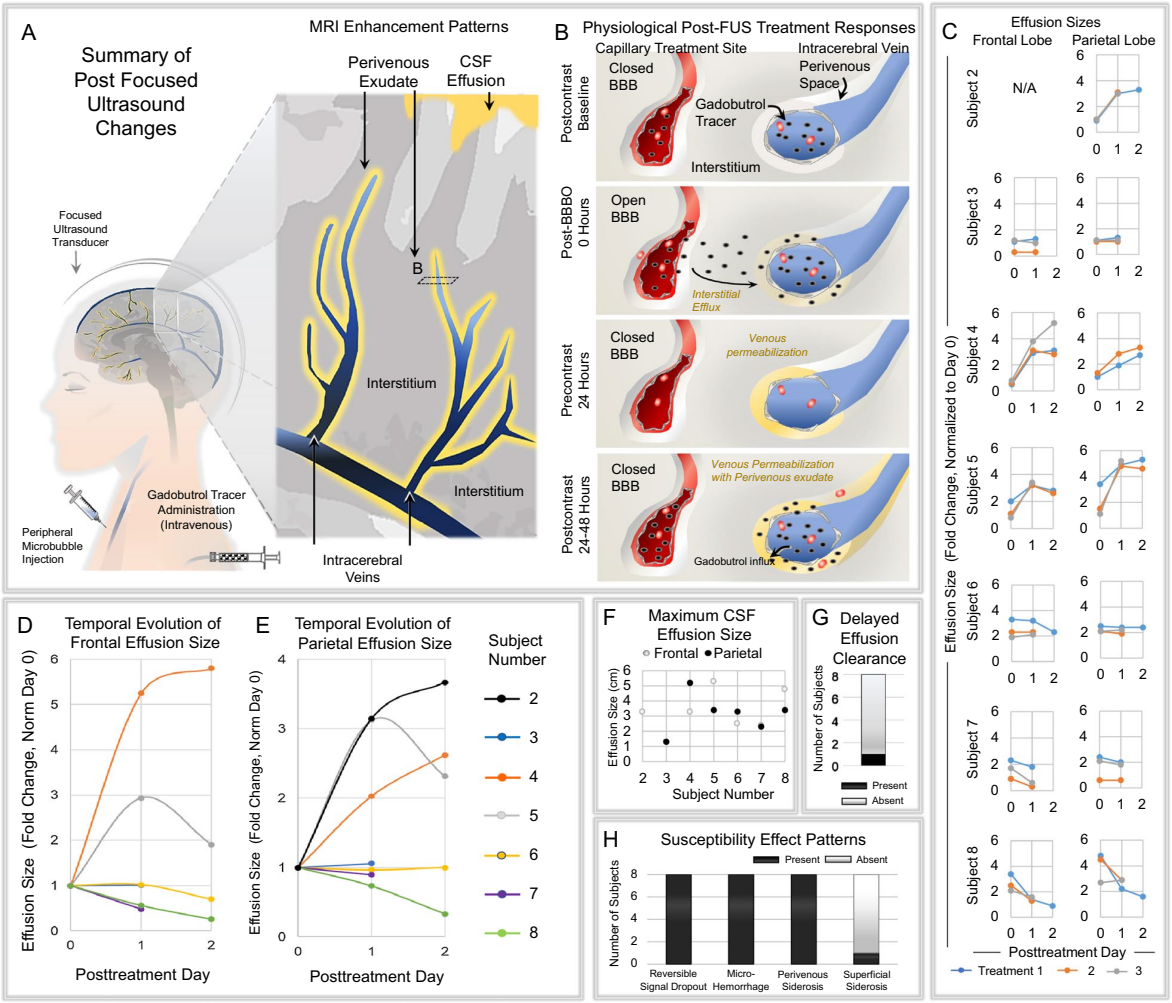
Summary of post FUS MRI effects and temporal progression of CSF effusions. Schematic diagram showing MRI enhancement patterns in the acute post-treatment period. This includes a perivenous pattern and subarachnoid space pattern of contrast enhancement **A**. Schematic diagram showing intracerebral contrast tracer accumulation at various time points **B**. Plots showing individual sulcal effusion size progression (in cm) over 3 time points in two regions of 7 subjects **C**. Plots showing mean fold change in size of sulcal effusions (values normalized to day 0 post-FUS) in each individual, within frontal and parietal regions, respectively **D**–**E**. Maximum effusion size shown in two regions of 7 subjects **F**. Number of persons with and without delayed effusion clearance is summarized in **G**. Plots showing number of treated subjects with various susceptibility effect patterns **H**

Complete clearance of contrast tracer was observed at all treated parenchymal brain sites of 8 of 8 persons (100%) within 24 h post sonication (Fig. 2E). No retained extraparenchymal contrast was visualized at the 24 h time point on T1-weighted images in any individual. Interestingly, contrast tracer was found to distribute in the perivenous regions but not throughout the interstitium of the sonicated brain volumes after repeated intravenous gadolinium-based contrast agent administration at 24–48 h following FUS (Fig. 3). These imaging findings indicate closure of the capillary BBB and permeabilization of intraparenchymal and extraparenchymal cerebral venous structures in 8 of 8 subjects (100%). Capillary BBB closure was indeterminate in certain cases on 24 h scans. Given these findings, it is concluded that BBB closure was achieved within 48 h post sonication in all cases. Linear and curvilinear patterns of contrast enhancement manifested within the brain parenchyma in the targeted frontal, parietal, and medial temporal lobes due to venous permeabilization that persisted beyond the time of BBB closure (Figs. 3, 4). The enhancement pattern was associated with discrete branching configurations in multiple cases (Fig. 4I) and invariably corresponded to regional cerebral venous architecture (Figs. 3, 4, 5, 6), as confirmed by SWI sequence analysis [22]. The venous permeabilization effect was visualized at 24 h post FUS intervention in all 8 subjects and, to a lesser intensity, at 48 h in 6 out of 6 subjects who underwent MR imaging at the 48 h time point (100%), and completely resolved in 8 of 8 individuals (100%) on T1-weighted imaging by one week (day 7 or 8). The temporal sequence of contrast enhancement effects was overall similar to the progression described previously (see Fig. 4 of Mehta et al., 2021 [20]) in the hippocampal region.

### Reactive CSF effusions persist up to 11 days after FUS treatment

Interestingly, CSF effusions were additionally observed following FUS-mediated BBB opening in 8 of 8 subjects (100%). The effusions manifested on post-contrast T2-FLAIR sequences as sulcal and CSF space hyperintensity along the surface of FUS-targeted brain regions (Fig. 5B). This MRI feature is consistent with leakage of gadolinium-based contrast agent from the intravenous compartment and its dispersion into the CSF compartment. In some cases, the effusions could be seen focally centered around subarachnoid veins. The CSF effusions were not detectable on pre-contrast T2-FLAIR sequences but were consistently visible on post-contrast T2-FLAIR images after all treatments in all treated individuals following BBB disruption. In 7 out of 8 subjects (88%), active effusions (i.e. new leakage of gadobutrol from the venous compartment) persisted for up to 7 days (after treatment 3; See Fig. 2B). In a single subject, the effusions may have persisted for up to 8–11 days. In this individual, pre-contrast T2-FLAIR imaging acquired on day 8 post FUS (following treatment 3, at the time point shown as red circle in Fig. 2B) demonstrated residual sulcal hyperintensity along the treated frontal and parietal lobes (Additional file 5, middle panel) suggesting delayed clearance of effused contrast material. The effusion completely resolved on subsequent follow up MRI (Additional file 5, lower panel). In all other subjects, pre-contrast T2-FLAIR images routinely exhibited rapid and complete clearance of effused sulcal contrast tracer within 24 h on follow-up MRI scans.

**Fig. 7:**
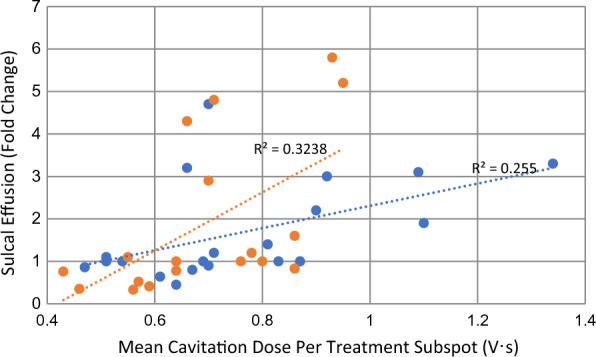
Correlation of sulcal effusion size progression with FUS cavitation dose. Scatterplot shows progression of sulcal effusion size (fold change at 24 h) values trend with delivered mean cavitation dose. Orange, frontal effusion measurements; Blue, parietal effusion measurements

The maximum effusion dimension varied from 1.2 to 5.3 cm in the eight participants. Effusion size in the frontal region averaged 1.6 cm on day 0 and 2.0 cm on day 1. Effusion size in the parietal region averaged 1.9 cm on day 0 and 2.6 cm on day 1. The temporal progression of effusions varied among individuals over 48 h following FUS treatment (Fig. 6C, D, E). Notably, effusion enlargement, detected upon repeat gadolinium-based contrast agent administration, at 24 h post-FUS trended with higher delivered FUS cavitation doses (Fig. 7; R^2^ = 0.3238 in frontal region; R^2^ = 0.255 in parietal region; R^2^ = 0.2211 overall). Variation in temporal effusion progression was not attributable to any specific difference in MRI protocol, including contrast agent administration timing, between the participants.

### Safety evaluation and post-FUS susceptibility effects

Signal changes manifested on blood-sensitive sequences at targeted brain regions following FUS (Fig. 5). Specifically, punctate foci of signal dropout were detected on T2* GRE and SWI sequences within targeted brain volumes (Fig. 5C) in 8 of 8 subjects (100%). These signal changes tended to correspond spatially with selected FUS target spots (Fig. 5A). At some FUS target spots, these signal changes were reversible and resolved completely by 48 h post FUS intervention (Fig. 5C). At other treatment sites, these T2* signal dropout changes persisted. In 8 of 8 subjects (100%), mild siderosis was also detected along the surface of cortical veins adjacent to targeted regions (Fig. 5D). This perivenous siderosis at least partially resolved in all patients. Susceptibility effects also manifested as focal mild superficial siderosis along cortical surfaces of targeted parietal brain regions in one subject (13%, Fig. 5D). No overt hemorrhage occurred in any individual. There was no mass effect associated with any region of hemorrhage or blood-breakdown products.

No serious adverse event was encountered by any trial participant. No hemorrhage or susceptibility effects were detected at any site remote from FUS-targeted brain volumes. No evidence of acute or chronic tissue injury, including ADC signal dropout to suggest cytotoxic edema, persistent parenchymal T2-FLAIR hyperintensity to suggest gliosis, or parenchymal volume loss was detected including at the regions of susceptibility effects and along sites of perivenous enhancement. Vasogenic edema occurred in the hippocampus of two subjects who received a higher hippocampal FUS cavitation dose, and was associated with local sulcal effacement in one patient, however these findings resolved completely without any residual parenchymal signal abnormality. No edema was detected in the frontal or parietal lobes of any individual. Quantitative segmentation analysis revealed no significant differences in volume of treated brain cortical regions (hippocampus, entorhinal cortex, and precuneus) at 60 days or 1 year following FUS, compared to baseline (ps > 0.3). T1 signal intensity returned to baseline at all treatment sites, without findings of parenchymal or extraparenchymal gadolinium retention or myelin loss. Study participants did not show any acute cognitive decline and formal neurologic and neuropsychological follow-up examinations have shown no unexpected clinical deterioration, after up to 48 months, compared to age-matched controls [18].

### Histology of perivenous spaces

**Fig. 8:**
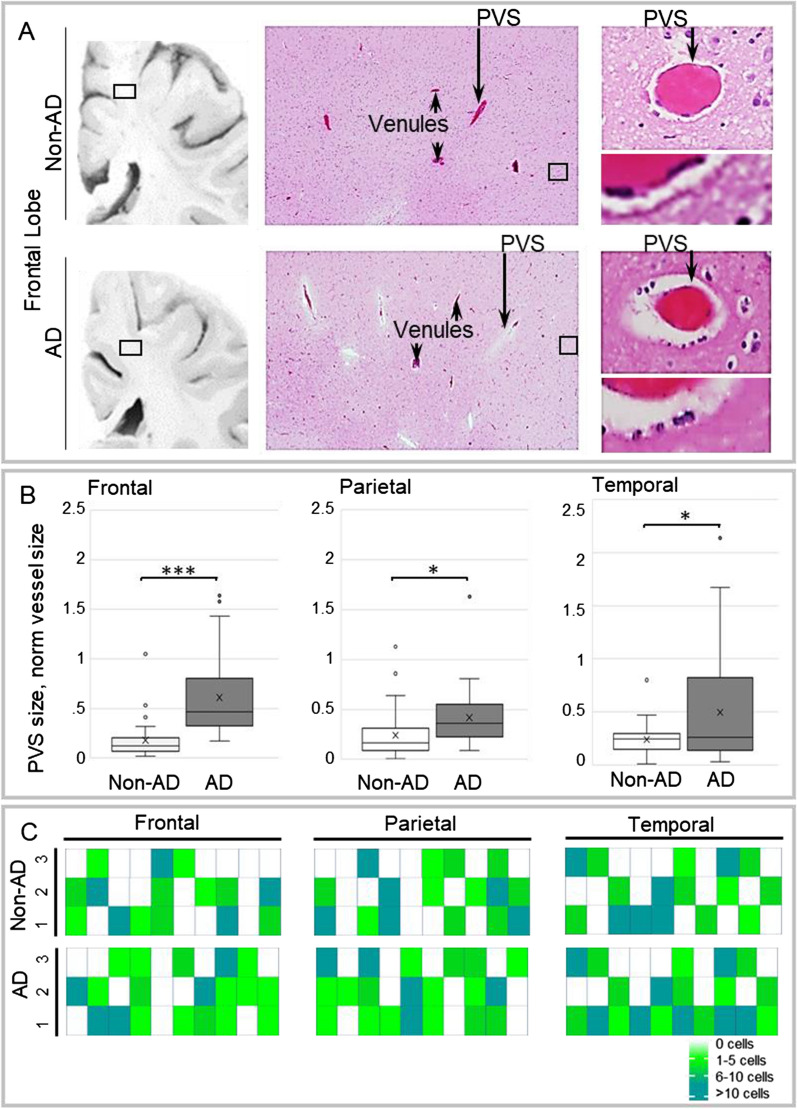
A network of perivenous spaces is identified on post-mortem brain specimens and corresponds anatomically with observed regions of MRI perivenous enhancement. Mild pitting is noted on coronal brain slices and is more prominent in persons with Alzheimer’s disease. **A** Boxed area in frontal subcortical region shows sampled region. On intermediate power microscopy images, perivenous spaces are noted. Perivenous spaces are shown to advantage on cropped images, which display mononuclear cells within the perivenous spaces (PVS). Plots summarize PVS size in frontal, parietal, and temporal regions of Alzheimer’s disease (AD) (n = 3) and non-AD (n = 3) decedents (**B**). Heterogeneity of immune cell density in PVS is shown in both AD and non-AD decedents (**C**)

Light microscopic analyses of frontal, parietal, and temporal brain regions revealed perivenous spaces, i.e., true conduits or cavities, situated between the brain parenchyma and the abluminal walls of intracerebral veins and venules (Fig. 8A). These spaces were present in all persons and their cross-sectional areas ranged from 1 to 214% of corresponding vessel area. Figure 8B summarizes perivenous space dimensions (normalized to vessel dimension) in frontal, parietal, and medial temporal lobe tissues. In all brain regions examined, perivenous spaces were significantly enlarged in subjects with AD relative to age-matched controls and contained variable numbers of mononuclear immune cells (Fig. 8A, right panel, C). These perivenous conduit spaces interconnected along the course of intracerebral veins and venules as they branched through cerebral gray and white matter parenchyma. The perivenous spaces formed cavities that were anatomically distinct from, but contiguous with, the brain interstium. No other discrete cavitary flow paths were observed on routine microscopy.

## Discussion

In this study, we explored the physiological responses to FUS-mediated BBB opening targeting various brain regions of individuals with early AD. Using in vivo MRI, we show that FUS-mediated BBB opening results in perivenous contrast tracer distribution patterns that support the existence of a brain-wide network of functionally compartmentalized spaces around cerebral veins. Concentration of gadobutrol tracer within this perivenous network suggests that these neurofluid channels likely serve as low-resistance pathways for intracerebral fluid flow and convective efflux in humans. Additional post-FUS MRI enhancement effects indicate prominent reactive venous permeabilization involving both intraparenchymal and downstream extraparenchymal veins. These findings, along with post-FUS sulcal effusions, shown for the first time in this study, correlate spatially and temporally with post-FUS immunological response patterns that have been documented in preclinical animal models [12, 13, 15, 24] and are notably consistent with a cerebral perivenous exudative response in humans.

Two prior studies documented contrast tracer accumulation along perivenous brain regions following clinical FUS procedures, however these prior works were limited in scope to the hippocampal region [20] and did not clearly or consistently delineate perivenous conduit spaces within the brain parenchyma [20, 25]. Here, we show more comprehensively and demonstrate in multiple cerebral lobes the presence of distinct perivenous spaces that interconnect within the brain and along meningeal veins to form a functionally compartmentalized fluid network. These data supplement the findings in prior publications [20, 25] and delineate a labyrinthine conduit system along the course of cortical, superficial medullary, and deep medullary veins as well as around draining extraparenchymal veins leading to the deep venous system and dural venous sinuses. The imaging findings suggest that perivenous flow occurs along a continuous efflux system that adjoins the brain interstitium with overlying meningeal tissue.

Differential hyperenhancement is demonstrated within this network upon BBB opening. The enhancement effects are unassociated with parenchymal edema or evidence of tissue injury. Moreover, the perivenous enhancement changes are invariably followed by rapid tracer clearance. This transient hyperenhancement phenomenon around cerebral veins is not explainable by tracer diffusion, pathological contrast enhancement, or retrograde flow of contrast tracer which has previously been suggested following FUS procedures [25]. Rather, the differential intraparenchymal tracer distribution pattern suggests preferential flow of fluid and small solutes within a low-pressure system of compartmentalized and interconnecting spaces that parallel the superficial and deep cerebral veins. This interpretation is consistent with prior physiological experiments that have determined by mathematical modeling that the general extracellular compartment of mammalian brain imparts too high a resistance to allow for significant convective flow of interstitial fluid and that any significant convective flow must occur via lower resistance intraparenchymal channels [26]. Convective fluid flow through a system of interconnecting perivascular channels has been studied predominantly in rodent species, however, available data suggest that such a system is conserved and is perhaps better developed in higher species [20, 25].

Remarkably, the patterns of contrast agent pooling described here are reproducible across persons and across brain regions, though the subtle fluid shift is not appreciable in preclinical models nor by postmortem histology in humans. While the perivenous tracer distribution phenomena were not described prospectively following FUS in animal models, retrospective review suggests that they may have manifested in non-human primates [7]. Since the brains of higher mammals would be expected to have greater dependence upon convective flow for rapid clearance of proteins and larger molecular-weight particles that are poorly cleared by diffusion [27] the difference in brain volume between humans and lower mammals may likely account for the prominence of the imaging phenomena described here.

In this study, we further demonstrate reactive changes along the perivenous network following FUS. Enhanced venous permeability is shown along the entire course of this perivenous system, both within the brain parenchyma and along downstream meningeal veins following BBB opening. These imaging data suggest that fluid enters the perivenous network via two routes, both through efflux from adjacent upstream brain interstitium and via extravasation, or exudation, from the intravenous compartment. This proposed physiology differs, in part, from the proposed glymphatic hypothesis [28], which asserts that intracerebral fluid flow is driven by aquaporin-4 water channels expressed by perivascular astrocyte endfeet [28], a contention which has been widely disputed by other experts [29–34]. Our data suggest additional physiological mechanisms are involved in perivascular fluid shifts. The physiology depicted here parallels peripheral venous biology [35]. In extracranial regions, venous exudation is well documented to occur in response to active inflammation and trauma [35]. Here, in the brain, the venous permeabilization response shown on MRI notably correlates spatially and temporally with inflammatory response patterns that have previously been documented in animal models following FUS procedures [12, 24], being most prominent within the first 24 h post intervention and lasting up to 8 days. This study is the first to demonstrate post FUS CSF effusions, which occur in association with enhanced meningeal vein permeability and persist up to 11 days post intervention. Notably, heterogeneous effusion responses occurred among persons following FUS, being particularly pronounced in certain individuals. Our data suggest a trend between effusion response and FUS cavitation doses. However, more comprehensive and systematic investigation into the relationships of FUS parameter effects is necessary for future application of this novel drug delivery modality. Moreover, the observation of delayed effusion clearance in one of eight trial participants indicates heterogeneity in CSF clearance responses and suggests the need for future investigation into patient-specific factors influencing neurofluid dynamics in aging and neurodegeneration.

Notably, post-FUS effusions and T2* susceptibility effects share some features with amyloid-related imaging abnormalities (ARIA) that have been described following administration of anti-amyloid immunotherapies [36]. This observed characteristic of FUS-related imaging changes may suggest common underlying immune-related clearance mechanisms as an etiology for the MRI outcomes, including the T2* effects [20, 36]. However, specific features of FUS-related imaging changes may be useful in differentiating post-FUS effects from ARIA effects. As demonstrated in this study, susceptibility alterations due to FUS did not occur in non-sonicated brain regions in any treated individual. Secondly, post-FUS sulcal effusions were detected only on post-contrast T2-FLAIR imaging and were undetectable on pre-contrast T2-FLAIR sequences. Additionally, while post-FUS effusions were sometimes extensive, they were only found along FUS-targeted brain regions and did not occur elsewhere in the cranial cavity.

Limitations of the present study should be noted and include small sample size of eight participants who underwent treatment at a single institution and were part of an ongoing multicenter clinical trial. Future studies assessing FUS imaging responses in larger cohorts are needed. Expanded datasets are also necessary to draw more definitive conclusions on possible cavitation dose-related responses and to understand influences of specific sonication parameters, microbubble factors, and individual patient-related and comorbidity factors on physiological effects of FUS-mediated BBB opening, as well as differences in patterns across brain regions. Furthermore, MRI data presented here were acquired serially, but at fixed time points. Future MRI analyses incorporating real-time dynamic imaging would contribute to knowledge of post-FUS intracerebral fluid flow in live humans and associated pathophysiologic abnormalities in AD [37]. Longitudinal and post-mortem studies including more detailed histologic analyses assessing treated patients may also elucidate specific perivenous changes and potential long-term and/or delayed consequences of FUS-mediated BBB opening in trial subjects.

## Conclusion

In this study, we show that transcranial FUS combined with systemically circulating microbubbles results in transient spatially precise BBB opening within multifocal intraparenchymal brain regions of persons with AD and characterize FUS-related brain imaging changes. These post-FUS imaging changes will likely be relevant to further understanding FUS-mediated clinical responses and pharmacokinetics and pharmacodynamics of novel neurotherapeutics. Post-FUS MRI reveals perivenous contrast accumulations that suggest the existence of a brain-wide perivenous fluid efflux route in humans. Permeabilization of veins along this network is shown here for the first time in various intracerebral regions of persons with AD. These clinical MRI features indicate that perivenous routes are sites of transient exudate formation that may facilitate immunological effluent clearance. Additional investigation is needed to further characterize these post-FUS perivenous reactions and to elucidate how modulation of this response by concurrent neurotherapeutic agent delivery may be leveraged to promote brain health in aging, Alzheimer’s disease, and other neurological disorders.

## Supplementary Information

**Additional file1: **Eligibility criteria.

**Additional file 2: **MRI sequence parameters.

**Additional file 3: **MRI images showing perivenous drainage around the inferior sagittal sinus. Axial post-contrast T1-weighted and T2-FLAIR images **A** and coronal post-contrast T1-weighted image, with zoomed section **B** show enhancement around the posterior pericallosal vein (arrowheads) and the inferior sagittal sinus after FUS-mediated BBB opening. Note ring-like enhancement surrounding the inferior sagittal sinus (arrow), **B**, enclosed within the inferior margin of the falx cerebri. The posterior pericallosal drainage site into the inferior sagittal sinus is shown (arrow), **C**. Findings are also demonstrated in Additional file 4 (video).

**Additional file 4: **Video showing perivenous drainage around the inferior sagittal sinus. Post-contrast T1 weighted MRI sequence demonstrates perivenous contrast enhancement in the parietal lobe as well as contrast enhancement around the posterior pericollosal vein (yellow arrow), draining around the inferior sagittal sinus (blue arrow) following FUS-mediated BBB opening.

**Additional file 5: **Delayed effusion clearance in one subject. Post-contrast T2-FLAIR images immediately following the third FUS session in a 73 year-old woman show right frontal and parietal effusions as CSF space hyperintensities (top panel). Pre-contrast T2-FLAIR images acquired on day 8 following FUS treatment demonstrate significant resolution of the sulcal contrast accumulation, however small volume sulcal contrast tracer remains present (arrows, middle panel). Pre-contrast T2-FLAIR images on subsequent MRI scan (3 months post FUS treatment 3) document complete clearance of the effusions (lower panel). This delayed clearance following the third treatment was despite complete clearance of effused tracer material and resolution of active effusions documented on day 11 post treatment 1 in this individual (Day 8-13 MRI was not acquired following treatment 2 in this patient).

## Data Availability

Data generated or analyzed during the study are available from the corresponding author upon reasonable request.

## Abbreviations

AD: Alzheimer’s disease
ARIA: Amyloid-related imaging abnormalities
BBB: Blood–brain barrier
CSF: Cerebrospinal fluid
FUS: Focused ultrasound
ISF: Interstitial fluid
