# Supplementary material for: Ultrasound-mediated blood–brain barrier opening uncovers an intracerebral perivenous fluid network in persons with Alzheimer’s disease
Source: Fluids Barriers CNS. 2023 Jun 16;20:46. doi: 10.1186/s12987-023-00447-y (PMC10276371; doi:10.1186/s12987-023-00447-y)
Supplement: Supplementary file 1 — Additional file1: Eligibility criteria. [file 12987_2023_447_MOESM1_ESM.docx]

**Supplemental file: Eligibility Criteria**

**Inclusion Criteria:**

1. Male or Female between 50-85 years of age

2. Able and willing to give informed consent, or has delegated this to Legally

Authorized Representative

3. Probable AD consistent with NIA/AA criteria

4. Modified Hachinski Ischemia Scale (MHIS) score of <= 4

5. Mini Mental State Exam (MMSE) scores 18-26. The MMSE adjusted scoring

instructions will be followed, as applicable.

6. Short form Geriatric Depression Scale (GDS) score of <= 6

7. If receiving concurrent treatment with an AChEI and/or memantine, has been on the

medication for at least 3 months with a stable dose for at least 3 months prior to the

first Exablate BBB procedure.

8. Amyloid PET scan consistent with the presence of β-amyloid

9. Able to communicate sensations during the Exablate MRgFUS procedure

10. Able to attend all study visits (i.e., life expectancy of 1 year)

11. Ambulatory

**Exclusion Criteria:**

1. MRI findings:

● Active or chronic infection/inflammation

● Acute or chronic hemorrhages, specifically > 4 lobar microbleeds, and no

siderosis or macrohemorrhages

● Tumor/space occupying lesion

● Meningeal enhancement

● Intracranial hypotension

2. More than 30% of the skull area traversed by the sonication pathway is covered by

scars, scalp disorders (e.g., eczema), or atrophy of the scalp

3. Clips, shunts, or other metallic implanted objects in the skull or the brain and the

presence of unknown or MR unsafe devices anywhere in the body

4. Significant cardiac disease or unstable hemodynamic status, including:

● Unstable angina pectoris on medication

● Documented myocardial infarction within six months of enrollment

● Significant congestive heart failure defined with ejection fraction <40

● Subjects with unstable ventricular arrhythmias

● Subjects with atrial arrhythmias that not rate-controlled

● Cardiac pacemaker

● Uncontrolled hypertension (diastolic BP > 100 on medication)

● Patient has right-to-left, bidirectional, or transient right-to-left cardiac shunts

● Patients with relative contraindications to either DEFINITY® ultrasound contrast

agent or PET amyloid tracer including subjects with a family or personal history

of QT prolongation or taking concomitant medications known to cause QTc

prolongation and:

o QT prolongation observed on screening ECG (QTc > 450 for men and >470

for women)

5. History of a bleeding disorder, coagulopathy or a history of spontaneous

hemorrhage

6. Receiving anticoagulant (e.g. warfarin) or antiplatelet (e.g. aspirin) therapy within

one week of focused ultrasound procedure or drugs known to increase risk or

hemorrhage (e.g. Avastin) within one month of focused ultrasound procedure

7. History of a liver disease, bleeding disorder, coagulopathy or a history of

spontaneous hemorrhage

8. Abnormal coagulation profile (PLT < 100,000), PT (>14) or PTT (>36), and INR > 1.3

9. More than 1 non-strategic lacune or any larger than 1.5 cm

10. Known cerebral or systemic vasculopathy

11. Significant depression and at potential risk of suicide

12. A severity score of 2 or more on any of the ‘Delusions’, ‘Hallucinations’ or

‘Agitation/Aggression’ subscales of the Neuropsychiatry Inventory (NPI-Q)

13. Known sensitivity/allergy to gadolinium (Gadovist), DEFINITY or its components, or

florbetaben.

14. Known hypersensitivity to DEFINITY or its components.

15. Any contraindications to MRI scanning, including:

● Large subjects not fitting comfortably into the scanner

● Difficulty lying supine and still for up to 3 hours in the MRI unit or claustrophobia

15. Any contraindication to lumbar puncture for collection of cerebral spinal fluid,

including:

● Intracranial hypotension

16. Untreated, uncontrolled sleep apnea

17. History of seizure disorder or epilepsy which could be worsened by disruption of

the blood brain barrier.

18. Impaired renal function with estimated glomerular filtration rate <30 mL/min/1.73m2

19. Does not have a reliable caregiver in frequent contact with the subject and can

accompany the subject to the clinic and treatment or be available by telephone at

designated times. Participants living in retirement homes may be included. Caregiver

not willing to sign the Informed Consent Form.

20. Currently in a clinical trial involving an investigational product or non-approved use

of a drug or device or in any other type of medical research.

21. Respiratory: chronic pulmonary disorders e.g. severe emphysema, pulmonary

vasculitis, or other causes of reduced pulmonary vascular cross-sectional area,

patients with a history of drug allergies, uncontrolled asthma or hay fever, and

multiple allergies where the benefit/risk of administering DEFINITY is considered

unfavorable by the study physicians in relation to the product monograph for

DEFINITY.

22. Brain atrophy severe enough to limit targeting.

23. History of drug or alcohol use disorder who may be at higher risk for seizure,

infection, and/or poor executive functioning.

24. Positive human immunodeficiency virus (HIV) which can lead to increased entry of

HIV into the brain parenchyma leading to HIV encephalitis.

25. Potential blood-borne infections, which can lead to increased entry to brain

parenchyma leading to meningitis or brain abscess.

26. Known apoliprotein E allele (ApoE4) homozygosity, which has been found to be

associated with thinning of the blood brain barrier

**Additional Exclusion Criteria for Cycles 2 and/or 3 of Exablate BBBD:**

Subjects who have experienced an adverse event or serious adverse event as noted

below (1-3) may be exempted from further Exablate BBBD procedures and go directly

to the latter follow-up visits.

1. A significant cognitive decline or significant mood/behavioral change

2. A significant and unresolved neurologic impairment noted at the 14 day assessment

prior to the next Exablate BBBD cycle.

3. A significant MRI finding (i.e., overt cerebral hemorrhage or infarction). Note: Some

degraded blood products at/around the target are expected as SWI signal.
