# Supplementary figures and images for: Ultrasound-mediated blood–brain barrier opening uncovers an intracerebral perivenous fluid network in persons with Alzheimer’s disease
Source: Fluids Barriers CNS. 2023 Jun 16;20:46. doi: 10.1186/s12987-023-00447-y (PMC10276371; doi:10.1186/s12987-023-00447-y)

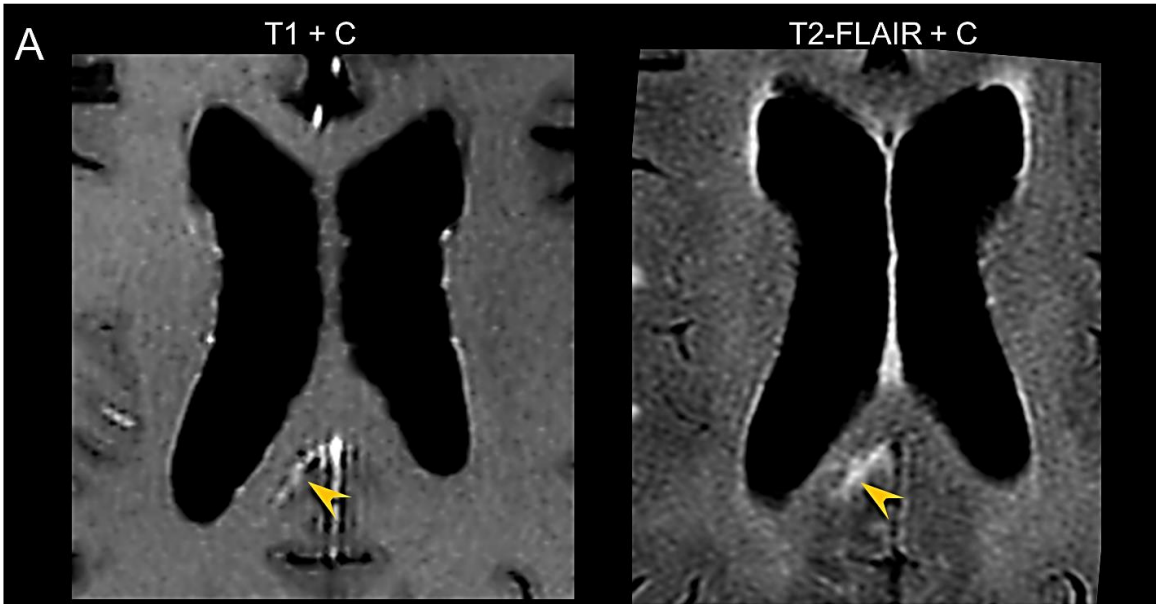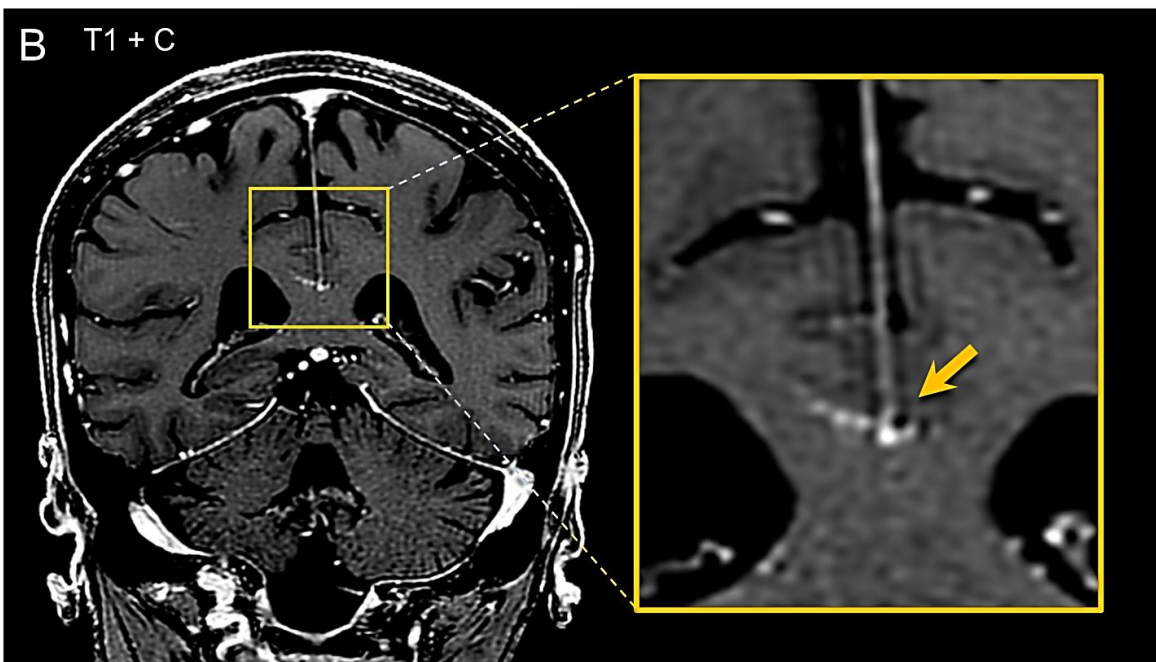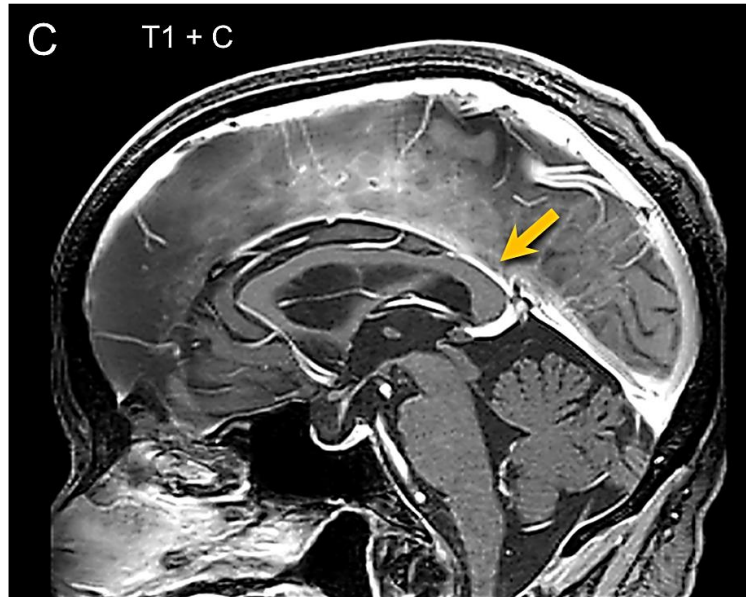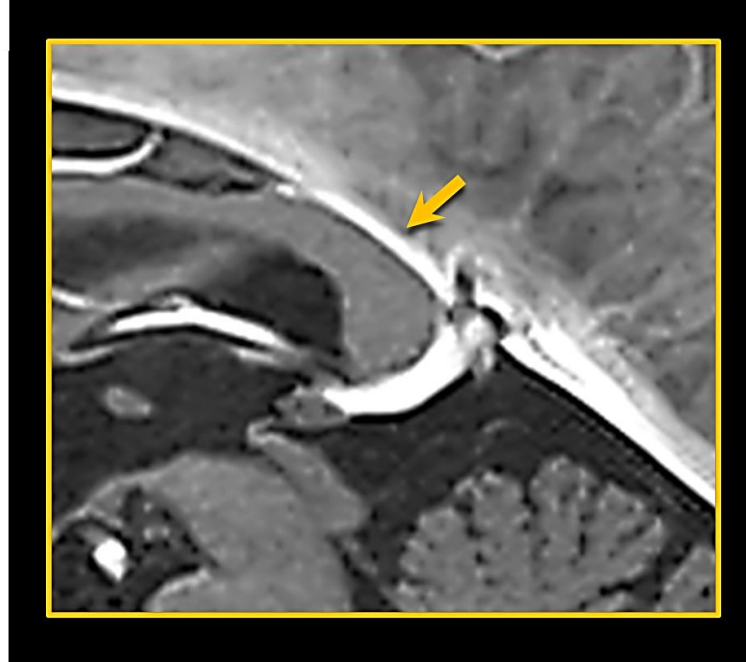

Supplement: Supplementary file 3 — Additional file 3: MRI images showing perivenous drainage around the inferior sagittal sinus. Axial post-contrast T1-weighted and T2-FLAIR images A and coronal post-contrast T1-weighted image, with zoomed section B show enhancement around the posterior pericallosal vein (arrowheads) and the inferior sagittal sinus after FUS-mediated BBB opening. Note ring-like enhancement surrounding the inferior sagittal sinus (arrow), B, enclosed within the inferior margin of the falx cerebri. The posterior pericallosal drainage site into the inferior sagittal sinus is shown (arrow), C. Findings are also demonstrated in Additional file 4 (video). [file 12987_2023_447_MOESM3_ESM.pdf]

3 Months Post FUS, Treatment 3

8 Days Post FUS, Treatment 3

Post FUS, Treatment 3

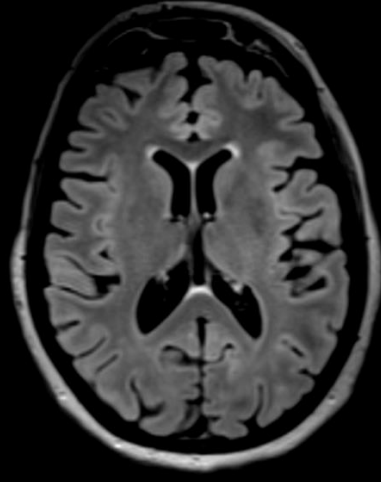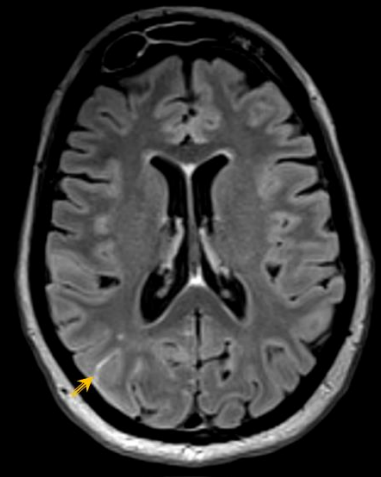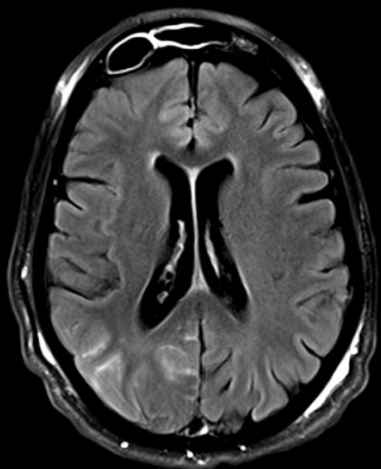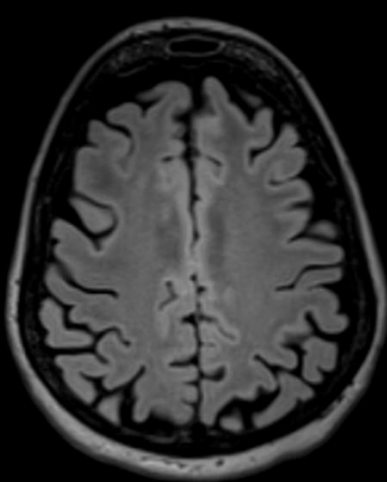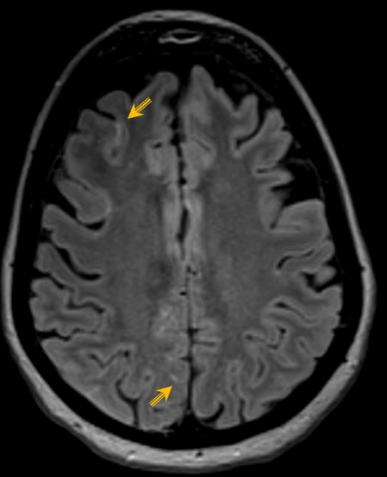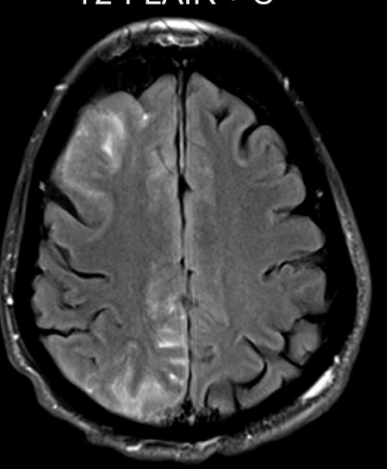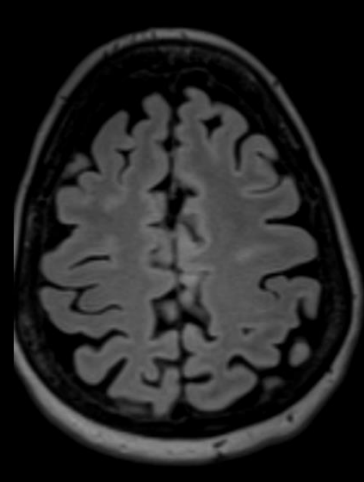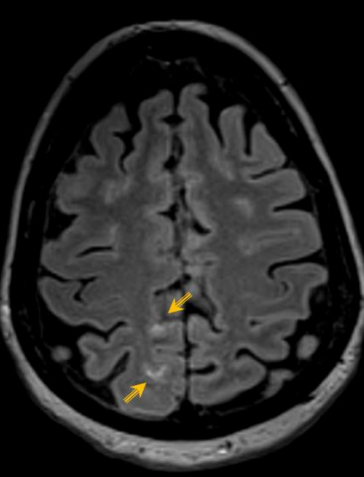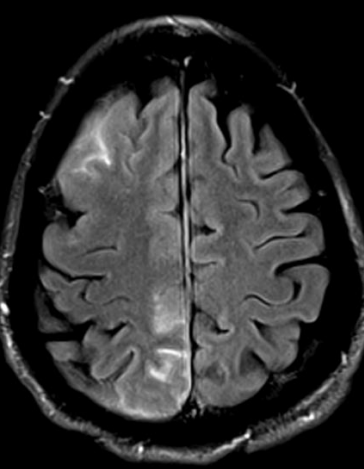

T2-FLAIR + C

T2-FLAIR

Supplement: Supplementary file 5 — Additional file 5: Delayed effusion clearance in one subject. Post-contrast T2-FLAIR images immediately following the third FUS session in a 73 year-old woman show right frontal and parietal effusions as CSF space hyperintensities (top panel). Pre-contrast T2-FLAIR images acquired on day 8 following FUS treatment demonstrate significant resolution of the sulcal contrast accumulation, however small volume sulcal contrast tracer remains present (arrows, middle panel). Pre-contrast T2-FLAIR images on subsequent MRI scan (3 months post FUS treatment 3) document complete clearance of the effusions (lower panel). This delayed clearance following the third treatment was despite complete clearance of effused tracer material and resolution of active effusions documented on day 11 post treatment 1 in this individual (Day 8-13 MRI was not acquired following treatment 2 in this patient). [file 12987_2023_447_MOESM5_ESM.pdf]
